# Catalysing change in health and medical research policy: an Australian case study of deliberative democracy to reform sex and gender policy recommendations

**DOI:** 10.3389/fpubh.2024.1522213

**Published:** 2025-02-12

**Authors:** Sue Haupt, Cheryl Carcel, Lily Halliday, Saraid Billiards, Lyn Carson, Kyle Redman, Scott Lappan-Newton, Karin R. Aubrey, Xander Bickendorf, Jane E. Bourke, Michael Buchert, Jessica Da Gama Duarte, Ayan Dasvarma, Thomas F. E. Drake-Brockman, Kerryn Drysdale, Stephen C. C. Dymock, Laura N. Eadie, Melanie Eckersley-Maslin, Moritz Falk Eissmann, James Fazio, Bridget G. Haire, Melinda Holder, Nicole Kleppe, Ken Knight, Jonathan Mauclair, Celine Northcott, Brian G. G. O. Oliver, Tracy A. O'Mara, Ken Pang, Steven Philpot, Tertia D. Purves-Tyson, Jacob Stewart-Olsen, Lauren Ursich, Natalia Vukelic, Marina H. Yakou, Bronwyn Graham, Severine Lamon, Rachel Huxley, Kelly Thompson, Keziah Bennett-Brook, Christine Jenkins, Zoe Wainer, Mark Woodward, Louise Chappell, Robyn Norton

**Affiliations:** ^1^The George Institute for Global Health, Women’s Health Program, Centre for Sex and Gender Equity in Health and Medicine, University of New South Wales (UNSW), Sydney, NSW, Australia; ^2^The George Institute for Global Health, Brain Health Program, UNSW, Sydney, NSW, Australia; ^3^Australian Human Rights Institute, UNSW, Sydney, NSW, Australia; ^4^Association of Australian Medical Research Institute, Melbourne, VIC, Australia; ^5^The newDemocracy Foundation, Sydney, NSW, Australia; ^6^Gauge Consulting, Otford, NSW, Australia; ^7^Pain Management Research Laboratories, Kolling Institute, Faculty of Medicine and Health, University of Sydney, Camperdown, NSW, Australia; ^8^Telethon Kids Institute, Nedlands, WA, Australia; ^9^Biomedicine Discovery Institute, Monash University, Clayton, VIC, Australia; ^10^Olivia Newton-John Cancer Research Institute, La Trobe University School of Cancer Medicine, Heidelberg, VIC, Australia; ^11^Anaesthesia and Pain Medicine, Perth Children’s Hospital, Nedlands, WA, Australia; ^12^Perioperative Medicine Team, The Kids Research Institute Australia, Nedlands, WA, Australia; ^13^Medical School, The University of Western Australia, Perth, WA, Australia; ^14^Institute for Paediatric Perioperative Excellence, The University of Western Australia, Perth, WA, Australia; ^15^Centre for Social Research in Health, UNSW, Kensington, NSW, Australia; ^16^Precision Cancer Medicine Theme, South Australian Health and Medical Research Institute, Adelaide, SA, Australia; ^17^Faculty of Health and Medical Sciences, University of Adelaide, Adelaide, SA, Australia; ^18^Peter MacCallum Cancer Centre, Melbourne, VIC, Australia; ^19^Sir Peter MacCallum Department of Oncology, The University of Melbourne, Melbourne, VIC, Australia; ^20^Department of Anatomy and Physiology, The University of Melbourne, Melbourne, VIC, Australia; ^21^Kirby Institute, UNSW, Kensington, NSW, Australia; ^22^Westmead Institute for Medical Research, Westmead, WA, Australia; ^23^Murdoch Children’s Research Institute, Melbourne, VIC, Australia; ^24^Monash University, Clayton Campus, Clayton, VIC, Australia; ^25^South Australian Health and Medical Research Institute, Adelaide, SA, Australia; ^26^The Woolcock Institute of Medical Research, Macquarie University, University of Technology Sydney, Sydney, NSW, Australia; ^27^QIMR Berghofer Medical Research Institute, Herston, QLD, Australia; ^28^Neuroscience Research Australia, Sydney, Australia; Discipline of Psychiatry and Mental Health, UNSW, Sydney, NSW, Australia; ^29^The Florey Department of Neuroscience and Mental Health, University of Melbourne, Parkville, VIC, Australia; ^30^School of Psychology, UNSW, Sydney, NSW, Australia; ^31^Faculty of Health, Deakin University, Burwood, VIC, Australia; ^32^Nepean and Blue Mountains Local Health District, Sydney, NSW, Australia; ^33^The George Institute for Global Health, Guunu-maana (Heal) Aboriginal & Torres Strait Islander Health, UNSW, Sydney, NSW, Australia; ^34^The George Institute for Global Health, Respiratory Program, UNSW, Sydney, NSW, Australia; ^35^Faculty of Medicine, UNSW, Sydney, NSW, Australia; ^36^Faculty of Medicine, Dentistry and Health Science, University of Melbourne, Melbourne, VIC, Australia; ^37^Public Health Division, The Victorian Department of Health, Melbourne, VIC, Australia; ^38^The George Institute for Global Health, Senior Professorial Unit, UNSW, Sydney, NSW, Australia; ^39^The George Institute for Global Health, School of Public Health, Imperial College London, London, United Kingdom

**Keywords:** public health and medical research policy, health and medical research equity, innovative methodology, evidence-based change, democratic policy reform, sex-gender

## Abstract

Revising public health policy based on new data does not happen automatically. This is acutely relevant to the now undeniable evidence that many diseases develop differently between the sexes and may also be affected by gender. Current health and medical practices across the globe generally fail to cater for sex and gender effects in common diseases. Inadequate policy frameworks to guide the comprehensive inclusion of sex and gender in research jeopardises scientific rigour and ultimately the practices they underpin. To ensure that Australian health and medical research is fit-for-purpose, we realised that potent initiatives would be necessary to expedite strategic reframing of thought and behaviour. Here we report on our innovative engagement of end-users for democratic self-determined policy reform to guide health and medical research, based on robust data. We draw upon our specific study to outline seven key steps that can be adopted to accelerate effective change, across a breadth of evidence-based initiatives to reform health policies.

## Introduction

Medical best practices are founded on pre-clinical and clinical trials data, which are assumed to be reproducible, statistically robust, and sourced from well-designed experimental testing of rational hypotheses. If data underpinning a procedure is not reliable though, patients are at risk of suboptimal health management. This is a danger if biological sex differences are not adequately considered in experimental models adopted in health and medical research. In human studies, this is exacerbated if gender is insufficiently factored into analyses.

Compelling evidence now indicates that health can be significantly influenced by a person’s sex and gender (1). Traditionally, sex has been reported female or male at birth, largely based on genitalia. Some people have innate variations in sex characteristics that do not align with features typically associated with their sex. In contrast to sex, gender is a socially constructed concept that includes: woman, man and many other terms that do not fit this binary, including gender diverse people (2). Gender identity may or may not align with sex. The relative disease risks for people with innate variations of sex characteristics, and trans and gender diverse people are under-studied (3).

Although environmental exposures can affect health, there are many disparities in health outcomes that cannot be adequately explained by external influences alone and these point to biological differences. For example, while healthier lifestyle habits are more common among women than men and likely contribute to extended longevity, these extra years are often overshadowed by poor health, for reasons that are not fully explained by exposures and behaviours (4). Discriminant health outcomes between the sexes are evident across major causes of death, including cardiovascular diseases (5), that encompass ischemic heart disease (6) and stroke (7); cancers of non-reproductive organs (8); and many other diseases across the globe (1). In addition, gender can be an important influence on health, and health equity is at risk when populations are inadequately included in studies (3).

Male disease symptoms have typically guided diagnosis and treatment of many diseases (9). There are many reasons for this asymmetry, including: higher prevalence of many diseases in males, often at younger age (10); medical practice and research historically being a prerogative of men (11); dismission of feminine emotions (12); the complexity of female genetics (where a double dose of X chromosomes in females involves complex regulation to equate the gene dosing of males with single X chromosomes) (13); and female biology and reproductive capacity (exemplified in reference) (14). But if sex and gender difference in diseases are overlooked, there is a risk that heath care outcomes will not be equitable (15).

Prompted by these concerns, governing and regulatory bodies in North America and Europe (16), have primarily adopted top-down tactics to introduce corrective measures. These include: guidelines formulated over the past 10–15 years by publishers [e.g., SAGER (17, 18)]; policies by funding agencies in Canada (19), United States, Europe (16); and recommendations of cultural change in the United Kingdom (20). Education has been introduced into some universities and research institutions (21); and Japan and South Korea have held forums to promote awareness and change (16).

The Canadian policy initiatives have been reviewed to evaluate effectiveness. Introducing policy without support proved poorly effective though and over time, auxiliary interventions were added to promote compliance (19). Inclusion of biological sex in research design and reporting in science publications has now risen from ~22% in 2010 to 83% in 2019, with many human studies also considering gender (19). The Canadian experience indicated that sector-wide behavioural change on this topic is accelerated when policy reform is supplemented by direct researcher engagement.

Australia (22) and numerous other countries lag behind North America and Europe regarding sex and gender policies in health and medicine. Australia has been slow to recognise the importance of sex and gender in health and medicine, and even slower to take action (22). The Australian research sector is in dire need of correcting its course to be appropriately fit-for-purpose. Delayed actions on the other hand, offer opportunities to tackle this situation, guided by the experience of others.

Since undertaking our initiative, the Australian National Health and Medical Research Council (NHMRC) and the Department of Health and Aged Care (that oversees the administration of the Medical Research Future Fund, MRFF) has responded to this void with an on-line statement published as recently as 2024 (23), encouraging consideration of sex and gender variables in the research they fund. Learning from the Canadian studies (19) though, it is evident that additional measures are necessary for rapid transformation of recommendations into practice. Our manuscript outlines a creative scheme for policy revision to drive fundamental shifts in the mind-set and operation of discovery science- and clinical-researchers, in response to new evidence.

## Methods

### Project aims and rationale

Our novel approach to fostering policy transformation has been to engage the target sector at its grass-roots, and in turn leverage their influence to catalyse change across all levels. The overall goal of our specific initiative is to ensure genuine and strategic consideration of biological sex, throughout all stages of discovery studies and clinical trials; with gender also addressed where relevant; leading ultimately to advanced and equitable patient care. Our immediate aims from our 2023 initiative were: first, to engage representative stakeholders from across the Australian health and medical research community; to second, facilitate their drafting of evidence-based guidelines for self-directing routine considerations of biological sex and gender in medical research; that would third, accelerate transformative practice across the sector.

We reasoned that by consulting with people who would be impacted by the proposed changes, and integrating their peer feedback, we would achieve quality decision-making, that would foster trustworthy and constructive change. We conjectured that exposure of end-users to evidence would drive rational transformation in thought and behaviour. We expected that this would be far more effective than tokenistic compliance to satisfy bureaucratic regulations that are frequently perceived as stumbling blocks. Our base-up, guideline formulation strategy is distinct, yet complementary to the important, top-down approaches adopted by regulatory bodies in Australia (23), and other countries (16).

### Design

We identified seven steps to fast-track evidenced-based policy change, to transform practice. Adopting a deliberative democracy approach was core to our course of action. The deliberative democratic process in this context requires facilitated: recruitment of representative participants from a target population; participant exposure to diverse angles of the on-topic evidence base around a chosen remit; thoughtful and open engagement with the subject matter; inclusive, reasoned, on-topic discussions; in advance of consensus decision-making around chosen aspects of the selected topic (24) (Table 1).

**Table 1:** Seven steps to effective policy formation through deliberative democracy.

Identify a topic for change, establish functional leadership and secure resources to enable the process Build a team of organisers and facilitators Select a target population, recruit panel members via lottery and build trust and rapport Expose the evidence-base Deliberate and democratically draft policy Endorse and disseminate the policy among the target population Reinforce and evolve for endurance

Equally, adopting this process is applicable for deciding whether there is sufficient justification for change and if so, to what extent, and how to propel the process. This approach has broad reach and could be applied to any evidence-base underpinning practice. Our method is a fresh, bottom-up strategy, that cultivates change by engaging sector stakeholders in policy design workshops where they are exposed to robust data.

We capitalised on our specific Australian experience to formulate this universal blueprint for self-guided, fact-centred governance reform. We understand this adaption of the democratic deliberative process is a first-in-kind approach to overhauling sex and gender policy in health and medical research. We have distilled our specific experience into a sequence of actions that have wide scope for reforming health policies. Each step is listed chronologically below with accompanying text boxes that outline specific details from our Australian prototype of sex and gender policy reform.

**Step 1: Identify a topic of change, establish functional leadership, and secure resources to enable the process**. Recognising a matter that warrants consideration for priority reform is foundational to a successful first step. Key considerations are scoping the nature of the problem; the scale of the need; and the breadth of interest across stakeholders. Establishing functional leadership and sourcing adequate funding is crucial to achieving change.

**Step one: of the Seven Steps of Deliberative Policy Design.**

**Elaborated with Details of our Australian Initiative to Reform of Sex and Gender Policy in Australian Health and Medical Research**

**Identify a topic for change**: Clearly define the matter under scrutiny.

- Subject of the Australian example: Sex and gender policy in Australian health and medical research.

Our focus on sex and gender policy in health and medical research stemmed from our team’s exposure of a lack of adequate on-topic policy from Australian health and medical funding agencies, publishers (22) and universities (25).

**Establish respected and effective leadership**: Strategically partner to mediate the reform process.

- Our Australian joint venture fostered mutual collaboration in the interest of:

Health Equity: driven by The George Institute for Global Health (TGI): led by founding director Prof Robyn Norton; and

Human Rights Equity: orchestrated from the Australian Human Rights Institute UNSW: led by director Scientia Professor Louise Chappell

- With strategic engagement to the:

Health and Medical Research Sector in Australia: mediated by the Association of Australian Medical Research Institutes (AAMRI)-a non-government, not-for-profit coalition of allied interests that functions in sector consultation and coordination, advocacy, policy development, capacity building and information dissemination; led by CEO S.Billiards (SB), who independently prioritised this topic.

The chosen remit to address was: “What should AAMRI do to support the improved use of sex and gender in research practices and decision-making?”

**Secure resources to enable the process**: Gather financial support.

- Australian Resources: Anonymous philanthropic funding secured.

The following costs were specific to running our Australian Deliberative Policy Design;

Design and delivery of 3× 6 h workshop process: Gauge facilitator Scott Lappan-Newton led the facilitation for a discounted ~$30 k ex GST. Similarly, newDemocracy delivered ~$40 k-worth of process design, recruitment, preparation and delivery support, and project evaluation services for ~$15 k ex GST. The facilitators chose to offer their services at discount rates based on their expectation that they could contribute to making a difference for medical research, while demonstrating a new application of deliberative practice.

Costs of participant and speaker travel & accommodation: in our case, when the majority of the participant travelled from across Australia, for 2 in-person workshops -the total cost summed to ~$40 K. Such expenses will depend on location and accommodation requirements.

Venue hire and local support: required no additional payment as internal facilities were utilised.

**Step 2: Build a team of organisers and facilitators**. Building an operational team is essential for developing a professionally executed, scientifically-accurate programme driven by evidence, to achieve wide impact.

**Step two: Of the Seven Steps of Deliberative Policy Design.**

**Elaborated with Details of our Australian Initiative to Reform of Sex and Gender Policy in Australian Health and Medical Research**

**Build an operational team of organisers and facilitators**: Enlist professionals capable of coordinating meeting logistics, and mediators proficient in mediating deliberative discussions.

- Australian expert organisers and facilitators were carefully selected and engaged into an operational team to ensure seamless advancement of the goals.

Professional logistic workshop organisers were enlisted from academic and professional staff of the partnering institutes.

Professional expert facilitators were externally recruited to run the policy workshops - from Gauge Consulting (S.Lappan-Newton) and The newDemocracy Foundation (L.Carson and K.Redman).

**Step 3: Select a target population, recruit panel members via lottery and build trust and rapport**. Choosing a receptive cohort, performing unbiased selection of its representatives to participate in a deliberative panel and establishing courteous rules of engagement, are fundamental elements to this process. In recruiting the panel members, it is important that the overall goal and the process is explained, together with on outline of the expected level of time commitment. It is relevant to explain that the process involves a ‘Deliberative Panel’ approach that is a creative adaptation of a ‘Citizens Jury’, with the goal of facilitating end-users to self-impose policy reformation. This contrasts the more familiar consultative approaches adopted by researchers to inform [as reviewed (24)].

From the outset it is crucial to establish respectful interaction protocols both between participants, and towards the data to be delivered from a range of sources across the workshops. Civil and reciprocal engagement across all discussions, are key to successful working dynamics. This is vital to open inquiry.

**Step three: Of the Seven Steps of Deliberative Policy Design.**

**Elaborated with Details of our Australian Initiative to Reform of Sex and Gender Policy in Australian Health and Medical Research**

**Select a target population**: Identify the community relevant to the elected topic.

- The Australian health and medical research sector was the designated group, and partnership with AAMRI enabled access to 58 institutes, compromising 50% of the entire community. Teaming up with AAMRI is a crucial advance towards our long-term vision to catalyse change across the entire Australian medical research sector.
- The success of our recruitment process was reliant on the high regard that AAMRI has fostered among its affiliates. Panel members were drawn from the institutes who responded to the requests of AAMRI’s CEO for nominations of participants. A clear request for diverse participants was conveyed during the recruitment process and this was respected. Candidate participants were made aware of the time obligations to engage in this process and the >90% continuation of panel members to completion reflects the high level of enthusiasm and commitment to this process and the topic.

**Recruit panel members via lottery**: Selection of representatives of the target population using non-biased methods.

**Figure 1:**
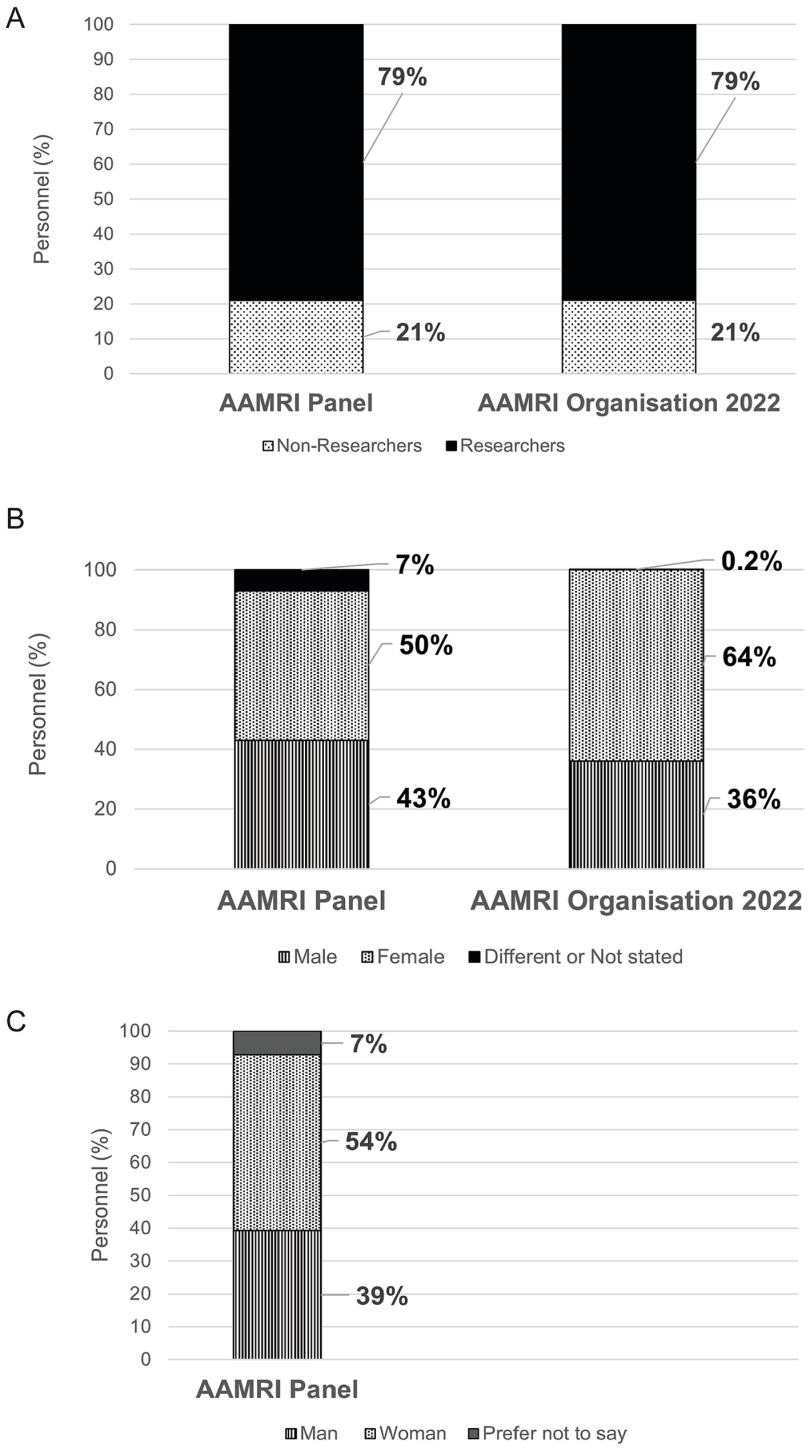
Demographic of AAMRI constituent organisations and the AAMRI workshop panel members. Membership of the deliberative panel was chosen to reflect the demographic of AAMRI members including researchers and professional staff **(A)**; and for sex **(B)** with gender also reported **(C)**.

- A 30-member panel of representative end-users from AAMRI stakeholders was sampled by the professional facilitators through a lottery draw. The selection was designed to randomly select candidates who descriptively represent AAMRI’s demographic (using a stratified random selection algorithm—deposited in GitHub, https://newDemocracy-Foundation.github.io/selection-tool/). Panel members were sampled for descriptive alignment with the overall demographic profile of AAMRI members with regard to: workplace role, sex, and gender [as reported in 2022 (26); Figures 1A–C]; with additional characterisation of research area, age and career stage recorded (Supplementary Figure 1). The starting candidate pool comprised nominees from 12 institutes that responded to a request to the directors of all AAMRI-affiliated institutes to each nominate 10 diverse staff members.

The panel members were informed that the set goal was: to undertake informed decision-making, for drafting policy recommendations to guide improved research practices, for all AAMRI members. From the initial panel members, 28 continued until the end of the entire process. All panel members actively were active participants in the preparation of this manuscript and are consented authors in this manuscript.

**Build trust and rapport**: Rely on facilitators to establish confident and respectful working relations, with civil engagement dynamics throughout the process, within a safe environment to improve chances of success.

- Professional facilitators established a culture of courteous and professional communication from the outset, encouraging open engagement and non-confrontational topic exploration between panel members and with presenters and introduced material.

**Step 4: Expose the evidence-base.**

Exposing scientifically accurate, objective and comprehensive data is the crucial component that underpins the quality of the drafted guidelines. Ensuring the availability of reliable, on-topic material from across a spectrum of perspectives, is central to exposing an evidence-base that can be rationally evaluated, and in turn catalyse genuine and sustained change in thinking and ultimately behaviour.

**Step four: of the Seven Steps of Deliberative Policy Design.**

**Elaborated with Details of our Australian Initiative to Reform of Sex and Gender Policy in Australian Health and Medical Research**

**Expose the evidence-base**: Deliver scientifically accurate material in an accessible format from a breadth of perspectives.

- Pre-Workshop 1 – An expansive information pack was delivered to panel members, setting the Australian situation into an international context, with added prompts for further subject exploration.

Workshop 1, 2 & 3 – Included panel discussions and breakout sessions with a breadth of topic experts: academic and clinical professionals; and individuals with diverse personal experience: including people with innate variations of sex characteristics and diverse genders, ages, and cultures -with maximum effort to recruit a breadth of opinions representing the widest level of intersectionality.

Specific Topics Addressed:

Workshop 1: In person interactive presentations on: Theory of change; Barriers and facilitators of change; Importance of sex and gender considerations at the bench and in analyses for health and medical research; Sex and gender relevance to medical diagnosis and treatment exemplified through stroke; One women’s heart disease journey; Lived experience of the importance of sex and gender to health, well-being and relevance to human rights.

To effectively engage with these topics, the larger panel was broken down into smaller breakout groups and allocated 10 min, per each speaker, to pitch questions to these experts. The panel members were asked to focus their line of questioning towards better understand the remit of: *What should AAMRI do to support the improved use of sex and gender in research practices and decision-making*? At the completion of these rotations, the panel members wrote down their thoughts around: *What should we stop doing? What should we start doing? What should we continue doing?* These were shared as a group, discussed, and emerging common ideas were noted and knowledge gaps identified. This information fed into Workshop 2.

Pre-Workshop 2: Written and recorded responses were circulated to fill information gaps identified in Workshop 1, including: Details of the on-topic commitments and actions initiated from AAMRI, the academic leads, and the Australian government health funders; Evidence from identified international sources that policy change has impact; Resources for terminology; Additional personal examples of poor health outcomes when sex and gender and other intersecting factors (i.e., ethnicity and age) are not properly considered.

Workshop 2: Additional requested perspectives presented and discussed included: Commitment of AAMRI president elect to the process; Policy initiatives from the Australian government funding agency NHMRC; Different perspectives of health issues facing nonbinary people; Influence of ethnicity on health in the context of sex and gender considerations.

In summary: Evidence was delivered by the team’s academic leads in advance of the first meeting, as a customised information pack that presented a breadth of pertinent peer-reviewed publications and discussion points. This was complimented over the workshops, by topic experts, and people with relevant knowledge, across a range of perspectives, including those with lived experience.

**Step 5: Deliberate and democratically draft policy.**

Deliberation in this context involves thoughtful reflection on the evidence base of a chosen topic, approached from various perspectives in a non-coercive manner. Thorough critical deliberation on available evidence is central to exposing biases, gaps in knowledge and suboptimal behaviours. Recommendations for solutions must be guided by evidence.

Concentrated review of robust evidence is in turn crucially partnered with democratic decision making and inform policy drafting in this process. Clear and effective deliberative democracy relies on thorough explanation of the principles of the process. Recruiting expert and experienced facilitators to introduce the process in a workshop format ensures professional delivery and expedites outcomes.

**Figure 2:**
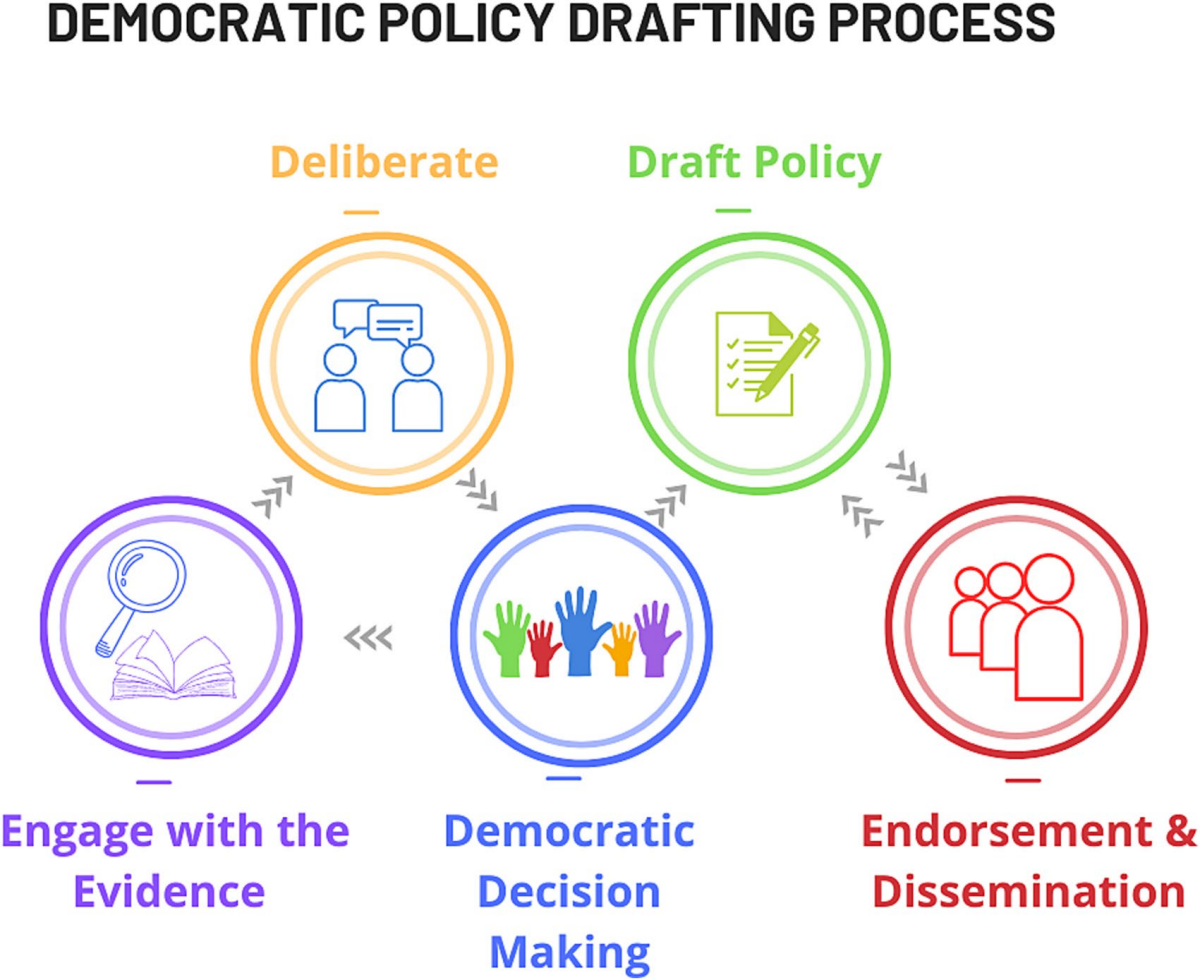
The cyclic processes of the deliberative policy drafting process.

Effective deliberation requires: dedicating time and attention by each participant to the process; embracing discussions and contributing constructive input; fostering functional spontaneous group dynamics to ensure all voices are heard and ideas adequately recorded and shared among the group. Productive outputs evolve from a repetitious cycle of topic engagement - deliberation - decision making; oscillating around an axis of divergence-emergence-convergence (Figure 2). Consensus on the proposals is crucial for democratic-decision making within the panel (24). Key elements for successful progression to consensus include: realistic expectations of drafted policy recommendations; willingness to listen to alternative views and compromise for consensus; and capacity to reach decisions democratically within the given timeframe.

A three-day immersive, deliberative process spaced at two-week intervals between meetings is a minimal model for achieving recommendation consensus. In person meetings are recommended but virtual elements, such as an on-line session, may also be considered. Extended programmes that are entirely in-person, involving a fourth day for expanded deliberation to reach consensus, are highly recommended as best practice for many applications.

**Step five: of the Seven Steps of Deliberative Policy Design.**

**Elaborated with Details of our Australian Initiative to Reform of Sex and Gender Policy in Australian Health and Medical Research**

**Deliberate and democratically draft policy:** Maximising policy drafting success requires clear explanation of the principles of the process of deliberative democracy: Engagement with the evidence; Deliberation upon it; Democratic proposal of changes. These activities are performed as a repetitious cycle to advance consensus and ultimately policy drafting (Figure 2).

- To effectively address the AAMRI remit, a three-day immersive, deliberative workshop, spaced at two-week intervals between meetings was adopted. In our case, the first and third workshops were in person and the second was virtual.

The guiding principles of the deliberative process were introduced to the panel in workshop 1 to equip them to contend with the evidence base. Prepared in this way, the panel members were equipped to identify holes in the evidence base and brainstorm deficits in practice. The academic leads worked to provide supplementary information by engaging additional topic experts in advance of the second workshop to feed the cycle of informed topic engagement and deliberation, to ultimately achieve consensus.

The evidence-base was further enhanced in the on-line workshop 2, with additional speakers recruited into interactive small-group discussions with the panel members. Panel members were divided into groups of four and each group rotated in turn with every speaker. Panel members were each asked to list their top two insights into the central AAMRI remit. Through interactive discussion with all the panel members together, these ideas were clustered into five topic areas for in-depth deliberation. The topics related to terminology deficits; implementation; accountability; quality research and education.

The panel was again broken-up into groups for on-line extended deliberation of the major challenges associated with each of the listed topics. Each group was assigned one topic and tasked with listing what they considered to be its most challenging aspects. Panel members were then asked to individually propose up to three ideas for possible actions for change around these topics, prompted by the statement—‘How might we address’—the identified challenge. Subsequent deliberation in assigned groups focused on developing these ‘crowd-sourced’ proposed actions into a series of refined, draft recommendations to mediate the desired changes. An important aspect of each group’s job was to represent the entire panel, not just their own perspective, when drafting recommendations.

At the end of Workshop 2, an initial preliminary draft of policy recommendations had been developed for review. Topic experts, and AAMRI and the academic leads provided brief written responses to the recommendations in advance of the final workshop—outlining their understanding of the recommendations, what they would do with that advice and asking further questions of the group—to aid them in finalising the recommendations. These were circulated to all panel members prior to Workshop 3. Panel members requested to complete an online survey at this stage, voting on how comfortable they were with each recommendation (according to a scale of: Love, Like, Live-with, Loath) and were requested to suggest tangible improvements.

In Workshop 3, the policy recommendations and comments were opened for further review and discussion in response to the topic experts in the final session. Panel members worked cohesively within the given timeframe to understand outstanding concerns and resolve these in updated versions of each recommendation. An advanced draft was democratically developed by the panel, building from small group discussions to panel-wide elections. To measure consensus, each recommendation was voted for majority approval prior to inclusion. Approval was set to 80% acceptance of each policy recommendation. A consolidated set of four policy recommendations were accepted by the end of the Workshop.

Achieving collective decisions was the task of the facilitators. Their professional expertise in mediating amicable consensuses relied on their capacity to: direct respectful discussions, establish a realistic pass rate to each democratic vote (set at 80% in each round), and introduce realistic time constraints. Setting reasonable expectations and defining time boundaries was crucial. Clear understanding of the benefit of reaching concrete outcomes was a strong motivation that led to the timely success of our process.

The facilitators were also responsible for supporting the process of leading the panellist members to grapple with the evidence-base to develop the final recommendations. The facilitator’s lack of expert knowledge on the chosen topic strategically avoided introducing pre-conceived biases, whether unconscious or conscious. The process would not have achieved such successful outcomes without the support of experienced deliberative facilitation. In summary: Throughout the first two workshops, in collaboration with the academic leads, the facilitators guided panel members to interrogate evidence, share knowledge and evaluate the current state of sex and gender considerations in Australian health and medical research. Gaps in knowledge and practice, barriers, challenges, and potential opportunities for improvement were uncovered. These formed the basis for the formulation of new policy recommendations that were produced from the third workshop to address the AAMRI remit.

**Step 6: Endorse and disseminate the policy among the target population**. Public backing and circulation of the policy reforms by the target organisation’s leader, across the breadth of the target population for wide-uptake.

**Step six: of the Seven Steps of Deliberative Policy Design.**

**Elaborated with Details of our Australian Initiative to Reform of Sex and Gender Policy in Australian Health and Medical Research**

**Endorse and disseminate the policy among the target population**: Secure approval and support from organisational leadership and circulate the policy to affiliates.

- Policy recommendations proposed by the panel members to guide the practice of AAMRI affiliates were submitted to the organisation’s CEO (SB) for consideration. Written support by the CEO on behalf of the organisation, including a feasible implementation strategy, was returned to the panel members and discussed. Once both parties were satisfied with the content, the policy recommendations were officially endorsed and disseminated among the member organisations for adoption. AAMRI CEO published on-line the recommended policy changes and the organisation’s steps towards catalysing change [Table 2; (27)].

**Table 2:** Policy recommendations.

AAMRI will advocate to MRIs and all sector stakeholders for visibility and awareness of the critical need for consideration of sex, sex characteristics, sex and gender variables in health and medical research (HMR). This is integral to quality and ethical research. AAMRI acknowledges that sex, sex characteristics and sex and gender variables are umbrella terms that have multiple unique and interrelated concepts that evolve in meaning and application; these applications vary across the breadth of health and medical research. We recognise that they are not static and that best practice would require ongoing participation by stakeholders and regular review. AAMRI will commit to developing a robust mechanism to respond to ongoing change and complexity in the above by coordinating collaboration and co-design methods with relevant community leaders, those with lived experience, and research experts to revisit the definition/terminology/ontology and application of these terms in health and medical research on a regular basis. AAMRI will advocate for appropriate inclusion of monitoring, benchmarking, and reporting mechanisms across its spheres of influence (such as members, universities, funding bodies, and journals) regarding sex, sex characteristics, and sex and gender variables in health and medical research applications, research conduct, and reporting of findings. There is currently a lack of knowledge of the application of sex, sex characteristics and sex and gender variables among researchers and the wider community, which acts as a barrier to high quality inclusive research. AAMRI will lead continued education efforts aimed at increasing knowledge and understanding of sex, sex characteristics and sex and gender variables in health and medical research to ensure sustained understanding of how and why to conduct high quality research.

**Step 7: Reinforce and evolve for endurance**. The need to fortify and adapt for endurance was evident from the Canadian experience (28), which had exposed that a set of rules without practical support and commitment will not lead to efficient implementation.

**Step seven: of the Seven Steps of Deliberative Policy Design.**

**Elaborated with Details of our Australian Initiative to Reform of Sex and Gender Policy in Australian Health and Medical Research**

**Reinforce and evolve for endurance**: Ongoing support is crucial.

- AAMRI has undertaken key initiatives for promoting genuine and ongoing implementation of the panel policy recommendations it has published.

First, AAMRI actively encourages panel members to act as champions for knowledge dissemination on this topic, within their own institutes and networks.

Second, AAMRI is engaged in raising sector-wide awareness on this topic.

Third, AAMRI is committed to long term change, through dynamic partnership with the Australian Centre for Sex and Gender Equity in Health and Medicine that was launched nationally in March 2024.

## Results

Democratically formulated policy that requires a pre-determined majority consensus is the important output of Step 5. In our prototype example, through collaborative efforts, the panel members generated policy recommendations that aim to systematically address the current deficiencies in the integration of sex and gender in health and medical research practices in Australia. Four specific measures are recommended to catalyse sector-wide change from the grassroots up (Table 2). These recommendations ultimately seek to enhance equitable health outcomes across populations. The essence of AAMRI’s commitments for change are to include sex and gender considerations in research; to dynamically review terminology to remain contemporary; to promote compliance through monitoring; and to introduce on-topic education to its constituent member organisations. Each policy recommendation was accompanied by a list of advised supporting actions (27).

## Discussion

Introducing organisational change is reputed to be challenging (29). Changing practice in medicine specifically is notoriously slow, and on average, translating a research finding to implementation takes 17 years (30). This predicts that early sex and gender policy initiatives by the Canadian Institutes of Health Research (CIHR) from 2010, and the National Institutes of Health (NIH) from around 2016 (16), are yet to fully benefit patients, leaving many practices still disproportionately catering to males [e.g., (31)]. A Canadian study identified primary barriers to change were: inadequate knowledge and skill; perception of irrelevance and lack of feasibility; deficient funding support; and insufficient culture at an institutional level (32). Investigation by our co-authors of Australian obstacles to sex and gender policy development and implementation, also identified a lack of knowledge and skills (25); and funding deficiencies. An uneven availability of high-quality evidence-based research was also recognised as an impediment to change. This has arisen at least in part, from systematic biases in existing studies, caused by the failure to adopt representative inclusion on the basis of sex and gender (33). Additional impediments relate to: inadequate training and tools; a paucity of leadership to champion change; ill-defined terminology (25); a shortage of content experts, and few local examples of successful (33).

Our method offers a new way to speed-up policy reformation and overcome barriers linked to slow uptake. Our innovative variation of the Citizen’s Jury (24) approach directly empowers the impacted sector to script informed policies to lead responsible self-governance. This is based on open engagement with the available evidence and personal discovery of the shortcomings of existing practices. This pathway of research and analysis is the core business of academic scientists and is highly suited to their mind-set.

This process also offers a vigorous approach to delivering additional enablers. Overcoming resistance to change is an example. By fostering a respectful environment for open appraisal of multiple facets of an evidence base, participants are given the opportunity to rationally re-evaluate preconceived opinions. The concise output of our Australian process was a list of recommendations and supporting actions (Table 2) to surmount the barriers to change. The specific actions can be distilled down to: advocacy, definitive language, monitoring change and education (27). Dedicated Australian funding is required to achieve these changes.

Our work towards sex and gender policy introduction highlights the value of tapping into existing leadership networks to access sector representation: in our case AAMRI, comprising ~50% of the medical research sector in Australia. AAMRI provided a framework of compliance obligation from its members and a mechanism for its own ongoing commitment to lead enduring change - to ensure that initiatives are not static but are dynamically maintained and nurtured into the future. Notably, AAMRI will draw on the support of the newly launched Australian Centre for Sex and Gender Equity in Health and Medicine to meet its obligations (officially inaugurated March 2024). This concerted leadership for change on this front is timely, as the major government health and medical research funders in Australia issued a joint statement in mid-2024 (23) requesting that sex and gender be considered in its funding applications.

Beyond the immediate success of our policy draft, this initiative offers vision for application on several fronts. First, our pilot work is an excellent proof of principle of feasibility and encourages expansion to the remaining half of the Australian health and medical sector that are not under the AAMRI umbrella. Second, this approach is pertinent to other countries that are yet to advance policies on this topic. Third, these methods offer scope for adaptation for modifying a host of other issues where a solid evidence base provides a logical rationale for change, for public health and beyond. Australian researchers must rapidly and comprehensively embrace policy reform initiatives on sex and gender to drive game changing practices that are necessary for medical relevance. Adopting improved procedures is fundamental to medical advancement. This aligns with the nimble adoption of protocol transformation. Delays between discoveries and their implementation risk lives, which emphasises the benefits of accelerating translation. A direct advantage of our frame is that can be progressively moulded as new information comes to light, to keep regulations contemporary and relevant.

## Conclusion

This strategy of end-user policy design is a constructive approach to changing the culture of thinking and acting on a given topic. Exposing overwhelming evidence, hand-in-hand with outlining a path to reform, through personalising responsibility across the sector, is identified as a potent change catalyst. Our specific instance of introducing sex and gender policy recommendations exposes important general principles for innovating transformations that avoid bureaucratic stagnation and draconian threats. The outcomes of our specific initiative have driven policy directives that are currently cascading into practical, self-driven initiatives to spread the uptake of this paradigm shift. In addition to pioneering change, our work also emphasises the need for ongoing education, support and fostering to maintain momentum that sustains the uptake of altered behaviours.

## Data Availability

The original contributions presented in the study are included in the article/Supplementary material, further inquiries can be directed to the corresponding author.
