# Supplementary figures and images for: Catalysing change in health and medical research policy: an Australian case study of deliberative democracy to reform sex and gender policy recommendations
Source: Front Public Health. 2025 Feb 12;12:1522213. doi: 10.3389/fpubh.2024.1522213 (PMC11861193; doi:10.3389/fpubh.2024.1522213)

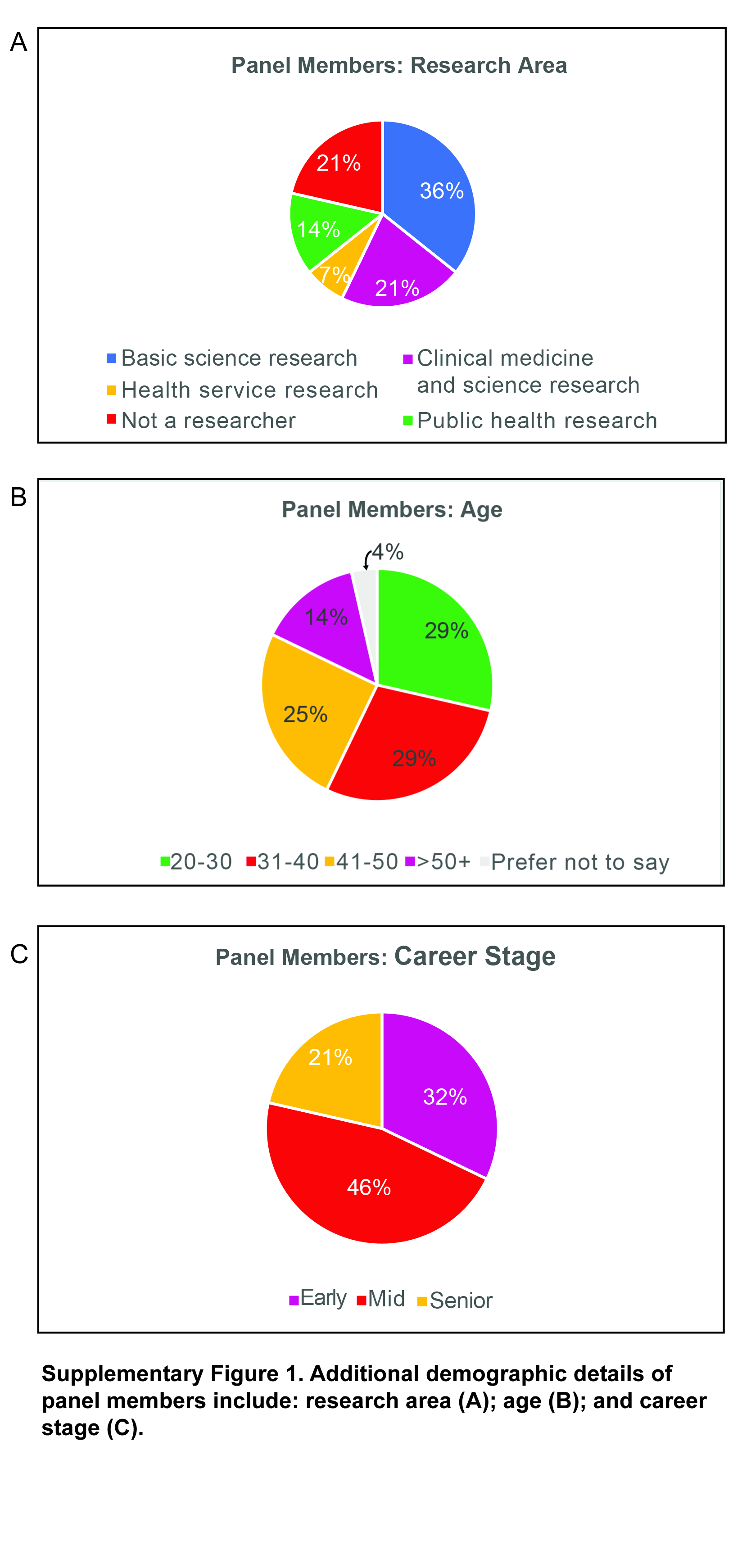

Supplement: Supplementary file 1 [file Image_1.tif]
